# Mechanical and Energy Absorption Properties of Porous Royal Water Lily Leaf Vein Cross-Sections Under Quasi-Static Axial Loading

**DOI:** 10.3390/biomimetics11050354

**Published:** 2026-05-20

**Authors:** Zhanhong Guo, Shuli Luo, Xiaowei He, Yichuan He, Caisheng Bai, Zhanhui Wang

**Affiliations:** 1 College of Mechanical and Electronic Engineering, Tarim University, Alar 843300, China; zhguo24@mails.jlu.edu.cn (Z.G.); 120140001@taru.edu.cn (X.H.);; 2 Key Laboratory for Bionics Engineering of Education Ministry, Jilin University, Changchun 130022, China; 3 Institute of Technology, Lanzhou Modern Vacational College, Lanzhou 730300, China; baicaisheng@163.com; 4 College of Chemistry and Chemical Engineering, Zhoukou Normal University, Zhoukou 466001, China

**Keywords:** Royal Water Lily Leaf vein, porous structure, gradient fractal, energy absorption, mechanical performance, response surface optimization

## Abstract

This study investigates the porous structure of Royal Water Lily Leaf vein cross-sections, integrating macroscopic structural observations, quasi-static compression experiments, and finite element simulations to systematically explore the influence of gradient fractal characteristics on mechanical performance and energy absorption behavior. First, the geometric features of the vein cross-sections were extracted through macroscopic measurements, and a parametric model incorporating key variables-porosity, pore ellipticity, and distribution density coefficient-was established. Single-factor analysis reveals that porosity plays a dominant role in determining the overall load-bearing capacity and energy absorption capability; pore ellipticity primarily affects local deformation modes and plateau-stage stability; while the distribution density coefficient significantly regulates the progressive and uniform deformation behavior. Subsequently, a multi-factor coupling model based on the Box–Behnken response surface methodology was developed to investigate the interactions among structural parameters. The results showed that the three variables exhibited significant synergistic effects rather than simple monotonic relationships. Within the investigated range, the optimized configuration (porosity = 30%, ellipticity = 1.6, distribution density coefficient = 1.5) achieved excellent comprehensive performance, with SEA = 115.75 J/kg, MCF = 248.2 N, and CFE = 0.445. Further analysis revealed that the porous vein structure does not exhibit strict self-similar fractal geometry but instead presents a gradient fractal characteristic with hierarchical progression and regional heterogeneity. During compression, the structure undergoes progressive collapse from the inner region outward, enabling staged load-bearing and efficient energy dissipation. These findings provide theoretical support and engineering guidance for the design and optimization of lightweight bioinspired porous energy-absorbing structures.

## 1. Introduction

Lightweight biological structures with excellent mechanical performance are widely observed in nature, among which the leaf structure of Royal Water Lily represents a remarkable example. Despite its large diameter, the leaf maintains a relatively low mass while sustaining considerable external loads, such as hydrostatic pressure and the weight of organisms. This superior load-bearing capability is primarily attributed to the complex and non-uniform morphological characteristics of its venation system [1]. In recent years, bio-inspired designs derived from the vein topology of Royal Water Lily have demonstrated promising applications in automotive engineering, railway transportation, and protective structures [2,3]. From a macroscopic perspective, the mechanical performance of the leaf is mainly governed by its well-developed venation network, particularly the porous architecture observed in the vein cross-sections. The venation system consists of radially distributed primary veins interconnected by circumferential elements, forming a structural configuration analogous to engineering truss systems [4]. Within this framework, a hierarchically distributed pore network is embedded, enabling the structure to maintain high stiffness and stability while significantly reducing material consumption and overall weight. However, existing studies have largely focused on morphological observations and bio-inspired conceptual designs, with limited attention paid to the quantitative relationship between key geometric parameters of the porous vein cross-sections-such as porosity, pore morphology, and spatial distribution-and their mechanical performance. Therefore, a systematic investigation based on parametric modeling is required to elucidate the intrinsic relationship between structural characteristics and macroscopic mechanical behavior, thereby providing a theoretical foundation for the design and optimization of bio-inspired porous structures.

In the field of plant biomechanics, researchers have extensively adopted multi-scale and multi-physics approaches that integrate mathematical modeling, experimental testing, and numerical simulation to systematically elucidate the relationship between structural morphology and mechanical function, spanning molecular, tissue, and organ levels. In terms of structural characterization and parametric modeling, Ma et al. [5] quantitatively analyzed leaf vein topological parameters and reported that vein number is not significantly correlated with the length of the main vein, whereas the total vein length exhibits a strong linear relationship with leaf perimeter. Li et al. [6] developed a three-dimensional composite model of sorghum stalks based on scanning electron microscopy and reverse reconstruction techniques, enabling cross-scale linkage from microstructural morphology to macroscopic mechanical behavior. Gangwar et al. [7] further advanced multi-scale characterization by combining microscopic imaging techniques (including micro-CT, optical microscopy, and transmission electron microscopy) with chemical analysis to quantitatively investigate the hierarchical structure and chemical composition of oat stems, spanning from cell wall to organ level. This work provided a solid foundation for establishing predictive models of mechanical performance. In addition, Mylo et al. [8] investigated the unique structural features of cactus branching junctions, analyzing their geometry and mechanical behavior, thereby offering new insights into the connection mechanisms of stable and detachable branching systems.

In experimental biomechanics, a wide range of mechanical testing approaches have been employed to characterize plant structures across multiple scales. Shah et al. [9] systematically reviewed various testing methods for herbaceous stems, including three-four-point bending and axial loading (tension, compression, and buckling), and provided standardized protocols and best practices, thereby establishing a methodological framework for plant mechanical characterization. Pierantoni et al. [10] developed a customized loading device compatible with micro-CT systems, enabling in situ three-dimensional visualization of leaf vein structures under compressive and bending loads, which offers critical insights into deformation mechanisms. For specific material systems, Ha et al. [11] conducted quasi-static compression tests on durian shells, quantitatively evaluating key parameters such as plateau stress, densification strain, and specific energy absorption under different loading directions. Lee et al. [12] investigated the creep and cyclic loading behavior of sweet sorghum stems, revealing pronounced viscoelasticity and time-dependent mechanical responses. Leblicq et al. [13] drew an analogy between the bending behavior of wheat and barley stems and thin-walled metallic tubes, distinguishing two sequential deformation stages—ovalization and buckling—and successfully applied classical mechanical models for tubes, achieving high predictive accuracy (R^2^ > 0.98). Furthermore, Speck and Spatz [14] performed comprehensive three-dimensional mechanical tests (longitudinal, transverse-vertical, and transverse-horizontal) on reed rhizomes. Their results indicated reduced anisotropy compared to hollow stems, attributed to short internodes and complex three-dimensional fiber arrangements, along with pronounced viscoelastic behavior under tensile and torsional loading. More recently, Anisimov et al. [15] proposed an improved inverse three-point bending setup, enabling high-sensitivity and wide-range measurements of elastic modulus in plant organs, thereby advancing precise mechanical characterization techniques.

In terms of numerical simulation and mechanistic analysis, various modeling approaches have been developed to investigate the mechanical behavior of plant structures. Ritzert et al. [16] constructed simplified fiber-based models derived from CT data and employed finite element analysis to study the mechanical response of the petiole–lamina transition zone. Peng et al. [17] analyzed the mechanical behavior of tree branching structures using finite element methods, systematically evaluating stress distribution and structural performance, and identifying key factors governing branch strength and stability. Regarding constitutive modeling, Song and Muliana [18] proposed a microstructure-informed constitutive model to describe the nonlinear and hysteretic responses of plant tissues under mechanical loading. The model assumes that a new internal network forms during deformation, which in turn influences the macroscopic mechanical behavior. Furthermore, Gangwar et al. [19] integrated microscale structural modeling with finite element simulations to predict the bending behavior of wheat and oat stems observed in wind tunnel experiments, revealing the multiscale origins of their macroscopic mechanical differences, such as the higher stiffness of wheat stems compared to oat stems. Kertész and Horváth [20] established a theoretical framework linking cross-sectional geometry to bending stiffness by comparing the second moment of area of triangular and circular stem cross-sections, providing valuable insights for structural optimization.

In terms of mechanical mechanisms and bioinspired implications, previous studies have revealed the high-efficiency mechanical performance of biological structures from both structural optimization and multiscale perspectives. Sun et al. [21] demonstrated through inverse optimization that a vein distribution approximating the golden ratio can significantly enhance the bending stiffness of leaves. Chen et al. [22] showed at the molecular scale that the functional gradient arrangement of cellulose microfibrils in plant cell walls effectively alleviates stress concentration and improves interfacial resistance to delamination. Song et al. [23,24] systematically investigated the structural composition and mechanical properties of sorghum and reed stalks from both macro-and microscale perspectives, highlighting the relationship between structural characteristics and static and dynamic mechanical behavior. In recent years, bioinspired energy-absorbing structures have attracted increasing attention due to their lightweight characteristics and excellent crashworthiness. For example, beetle-inspired hierarchical structures exhibit enhanced impact resistance and progressive deformation behavior through gradient geometric configurations [25], while nacre-like layered architectures achieve superior toughness and energy dissipation via staggered microstructural arrangements [26]. These studies demonstrate that biological structural optimization mechanisms provide important guidance for the design of advanced engineering energy-absorbing systems, establishing a multiscale framework for biomechanics and bio-inspired engineering and providing valuable insights into the efficient load-bearing and energy absorption mechanisms of natural structures.

However, existing studies have mainly focused on overall morphology, material constitutive behavior, or qualitative bioinspired concepts, while the quantitative relationship between the geometric configuration of porous vein cross-sections and their macroscopic mechanical performance in Royal Water Lily remains insufficiently understood. In particular, the independent and coupled effects of porosity, pore ellipticity, and pore distribution characteristics on compressive deformation and energy absorption behavior have not yet been systematically clarified. To address these gaps, the present study is based on principles of structural biomimetics and takes the central parenchyma region of Royal Water Lily leaf vein cross-sections as the biological prototype. High-resolution cross-sectional images were processed to extract key pore parameters, including pore area, major axis, minor axis, and spatial distribution. Subsequently, a parametric finite element model preserving the essential porous morphology was established and experimentally validated. Combined with single-factor analysis and Box–Behnken response surface methodology, the influences of porosity, pore ellipticity, and distribution density coefficient on compressive strength and energy absorption indices were quantitatively investigated. Compared with conventional uniform porous structures, the proposed gradient fractal-inspired configuration exhibits more stable progressive collapse and improved energy absorption efficiency. Therefore, this study establishes a quantitative framework linking biological structural characteristics with engineering mechanical performance and provides theoretical guidance for the design and optimization of advanced bioinspired energy-absorbing systems.

## 2. Materials and Experimental Scheme

### 2.1. Sample Preparation

**Figure 1:**
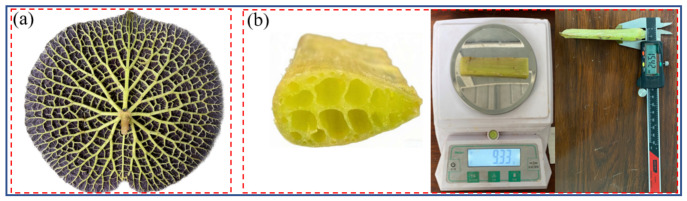
Royal Water Lily and test specimens (**a**) Royal Water Lily; (**b**) test specimens for macroscopic parameter measurement.

The Royal Water Lily leaves used in this study were collected in mid-August from the Lotus Pond in Dongshan Scenic Area, Suzhou, China. The sample was labeled as EL-1 (Euryale Leaf), as shown in Figure 1a. To minimize variability, all vein specimens were prepared using a consistent sampling and processing protocol. During sampling, the primary vein located in the central region of the leaf was selected as the object of study, as illustrated in Figure 1b, to ensure structural representativeness. Specimens with a length of 30 mm were cut along the axial direction of the vein for subsequent geometric characterization and mechanical analysis. A sharp cutting tool was used to prepare the specimens, ensuring smooth cross-sections without noticeable tearing or compression damage. This procedure preserved the integrity of the internal porous structure, allowing clear observation of pore distribution and providing a reliable basis for accurate measurement of pore geometry, including area, major axis, minor axis, and spatial position.

### 2.2. Measurement Method

**Figure 2:**
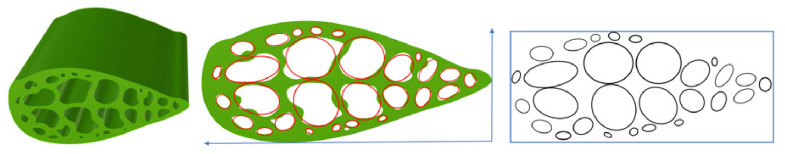
Measurement of the geometric parameters of a cross-section of Royal Water Lily Leaf vein.

To investigate the geometric characteristics of the porous structure in the vein cross-sections and their axial variation, systematic macroscopic measurements were conducted. All specimens were naturally dried at room temperature to eliminate the influence of moisture content on structural morphology. The cross-sectional shape of the vein is approximately elliptical. A reference line was defined across the center of the cross-section (from right to left) to establish a unified coordinate system for analyzing the fractal geometric characteristics of the structure. High-resolution images of the cross-sections were captured and processed using ImageJ software (Fiji) [27]. During image processing, grayscale conversion and binarization were applied to extract pore regions, followed by noise reduction to improve boundary detection accuracy. Based on the processed images, quantitative measurements of pore geometry-including pore area, major axis, minor axis, and spatial distribution-were obtained, as shown in Figure 2. Using these data, the variation of pore structural parameters along the axial direction of the vein (i.e., distance from the leaf center or reference point) was further analyzed. This provides a quantitative basis for identifying gradient fractal characteristics and supports subsequent bioinspired modeling.

### 2.3. Definition of Key Structural Parameters and Biological Prototype Basis

The selected structural parameters, including porosity, ellipticity, and distribution density coefficient, were derived from quantitative observations of the Royal Water Lily leaf vein cross-section rather than being arbitrarily prescribed. These parameters respectively characterize the lightweight level, anisotropic pore morphology, and regional gradient distribution of the porous structure, collectively representing the dominant macrostructural features of the biological prototype and therefore were adopted as key variables in the parametric analysis. Specifically, porosity was defined as the ratio of the total pore area to the entire cross-sectional area, quantitatively characterizing the lightweight feature of the structure. Ellipticity was defined as the ratio of the major to minor axis of each pore, reflecting the anisotropic geometric morphology observed in actual vein pores. Finally, the distribution density coefficient was introduced to describe the radial distribution relationship between pores in the inner and outer regions of the cross-section, based on experimentally observed non-uniform pore arrangements, thereby characterizing the gradient distribution feature of the porous structure.

### 2.4. Quasi-Static Compression Test of Leaf Veins

**Figure 3:**
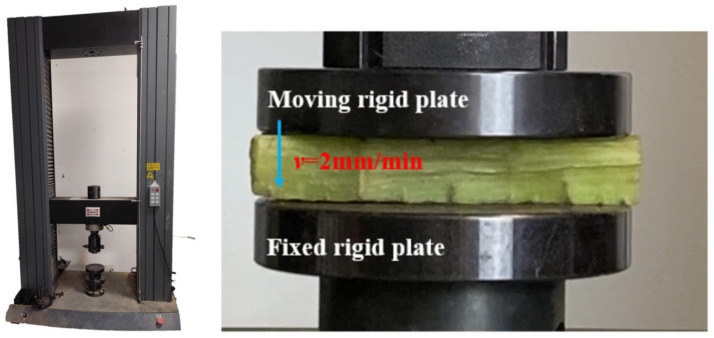
Axial quasi-static compression test on a leaf vein.

To validate the accuracy and reliability of the finite element model of the fractal vein structure of Royal Water Lily, quasi-static axial compression tests were conducted using a universal testing machine (Shenzhen Wan Test Equipment Co., Ltd., Shenzhen, China) [28]. The actual mechanical response of the specimens during loading was obtained and compared with numerical simulation results, thereby establishing the relationship between structural geometry and mechanical performance. As shown in Figure 3, to ensure loading stability and uniform stress distribution, a constant loading rate of 2 mm/min was applied during the test, with a total compression displacement of 15 mm. The load–displacement data were recorded in real time by a computer system, and the complete curves were exported for subsequent analysis [29].

### 2.5. Mechanical Performance Evaluation Criteria

To comprehensively evaluate the energy absorption performance of the thin-walled structure under quasi-static axial compression, this study adopts five key crashworthiness indicators: peak crushing force (PCF), total energy absorption (EA), specific energy absorption (SEA), mean crushing force (MCF), and crushing force efficiency (CFE) [30,31,32,33].

Total Energy Absorption (EA) primarily refers to the energy dissipated by energy-absorbing structures through their own deformation under impact, which can be calculated by integrating the load–displacement curve. The equation was as follows:

(1) $$EA=\int_{0}^{\delta_{\max}}F(x)dx$$

In Equation (1), *δ_max_* is the maximum effective compressive displacement; F(x) was the instantaneous crushing load at the compressive displacement x. The Specific Energy Absorption (SEA) represents the energy absorbed per unit mass of the energy-absorbing structure, defined as the ratio of the total energy absorption (EA) to the mass (M) of the energy-absorbing structure. A higher SEA value indicates better energy absorption performance. The expression was as follows:

(2) $$SEA=\frac{EA}{M}$$

The Mean Crush Force (MCF) represents the impact load per unit compression distance, defined as the ratio of the total energy absorption (EA) to the maximum effective displacement. A higher MCF value indicates better energy absorption performance. The equation was as follows:

(3) $$MCF=\frac{EA}{\delta_{\max}}$$

The Crush Force Efficiency (CFE) represents the stability of the impact load during the effective deformation process, serving as an indicator of the fluctuation in the impact load. The closer the CFE value was to 1, the higher the energy absorption efficiency. The equation was as follows:

(4) $$CFE=\frac{MCF}{PCF}$$

## 3. Macroscopic Structural Analysis of Leaf Vein Cross-Section

### 3.1. Distribution of Pore Area Along the Cross-Section

**Figure 4:**
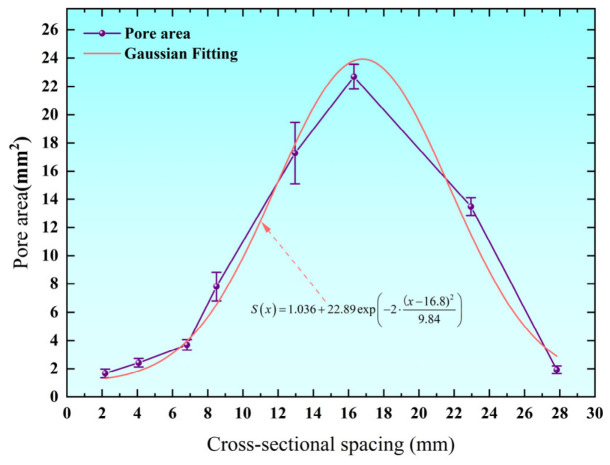
Distribution of pore area in leaf vein cross-sections.

As shown in Figure 4, statistical analysis of the pore area along the leaf vein cross-section reveals a pronounced nonlinear variation with respect to the distance from the starting point. Overall, the pore area exhibits a unimodal distribution, increasing gradually from the edge toward the central region, reaching a maximum value in the middle, and then decreasing toward the opposite edge. Specifically, the pore area is relatively small near the initial region of the cross-section, indicating a locally dense structure. As the distance increases, the pore area increases rapidly and reaches its maximum value of 20.824 mm^2^ at the sixth position, suggesting that the central region serves as the primary zone of pore development. Beyond this region, the pore area decreases significantly, and the structure becomes denser again toward the distal region. This distribution pattern indicates that the cross-section of the Royal Water Lily Leaf vein exhibits a graded porous architecture, characterized by a dense-sparse-dense transition from the edges to the center. The central region contributes to weight reduction through larger pores, while the denser edge regions enhance local stiffness and structural stability. This spatially varying porosity enables an effective balance between load-bearing capacity and energy absorption performance.

To further characterize the distribution of pore area, curve fitting analysis was performed on the measured data along the cross-sectional direction. The relationship between pore area S and spatial position can be expressed as follows:

(5) $$S\left(x\right)=1.036+22.89\;\exp\left(-2\cdot {\left(\frac{x-16.8}{9.84}\right)}^{2}\right)$$

The results demonstrate that the variation in pore area with distance is well described by a Gaussian function, with a coefficient of determination R^2^ = 0.96, indicating excellent agreement between the model and experimental data. The Gaussian distribution suggests the existence of a functional core region within the leaf vein cross-section, where the pore area reaches its maximum value and symmetrically decays toward both sides. This characteristic reflects an optimized natural material distribution strategy, providing valuable inspiration for the design of biomimetic porous energy-absorbing structures.

### 3.2. Distribution of Pore Geometric Scale (Major and Minor Axes)

**Figure 5:**
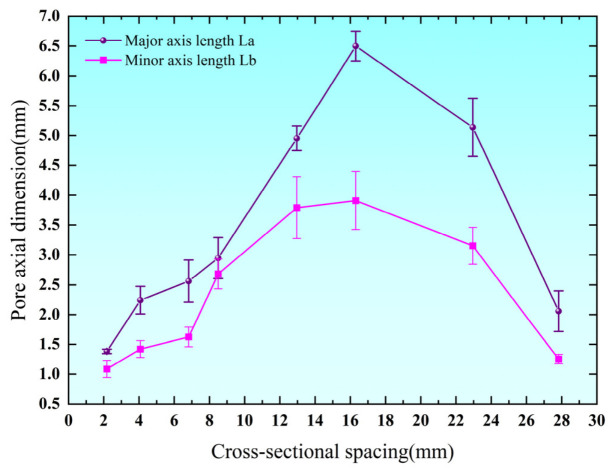
Distribution patterns of the major and minor axes of pore lengths in leaf vein cross-sections.

As shown in Figure 5, the pore geometric scale (major and minor axes) also exhibits a pronounced non-uniform distribution along the cross-sectional direction. Both the major and minor axes generally follow a trend of initial increase followed by a subsequent decrease with increasing distance from the starting point. Specifically, the major axis increases from 1.542 mm to a maximum value of 5.590 mm before slightly decreasing, while the minor axis shows a similar but less pronounced variation.

Based on the measured major and minor axes, the pore morphology was further characterized using the ellipticity and ductility parameters, defined as follows:

(6) α=La/Lb, β=Lb/La

**Figure 6:**
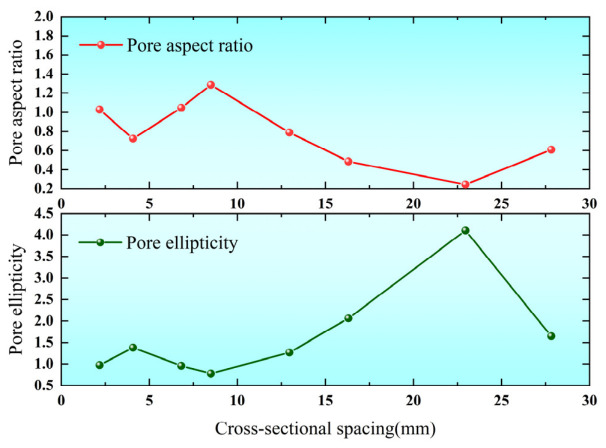
Patterns of ellipticity and ductility in the pores of leaf vein cross-sections.

The results in Figure 6 indicate that the pore ellipticity exhibits a distinct fluctuating trend along the cross-section. It increases rapidly from an initial value of 1.17 to 1.62, then gradually decreases to approximately 1.10, indicating a morphological transition from flattened pores to nearly circular shapes. Subsequently, the ellipticity increases again toward the distal region, suggesting a return to more elongated pore geometries. In contrast, the ductility parameter shows an opposite trend. These results demonstrate that the pore morphology across the leaf vein cross-section exhibits a spatially varying functional partitioning, reflecting a coordinated distribution of stiffness, energy absorption capacity, and reinforcement functions within different regions.

### 3.3. Distribution Characteristics of Overall Cross-Section Width

**Figure 7:**
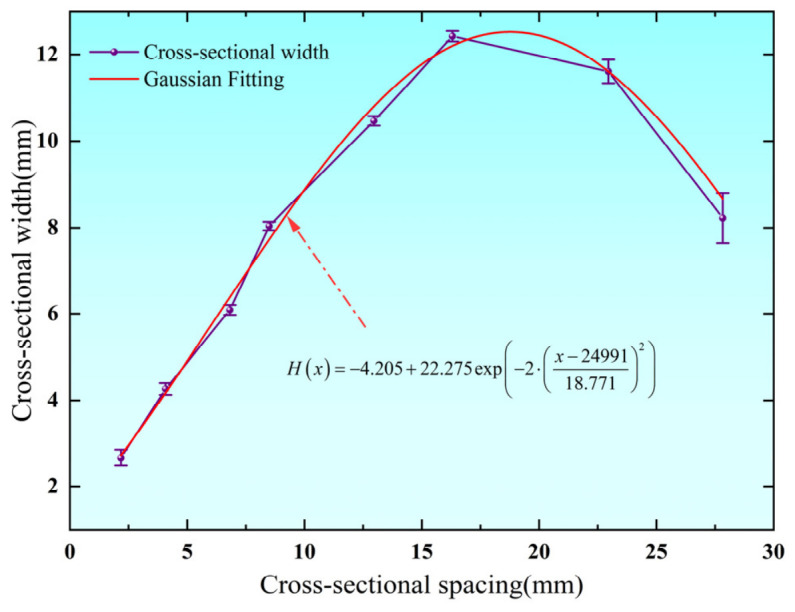
Distribution of leaf vein cross-sectional width along the cross-section.

As shown in Figure 7, the width of the leaf vein cross-section exhibits a pronounced asymmetric distribution along the longitudinal direction. The overall trend shows an initial increase from the starting point toward the central region, reaching a maximum value of approximately 12.42 mm, followed by a gradual decrease toward the distal end. This variation in width is highly consistent with the trends observed in pore area and geometric scale, indicating a strong coupling relationship between the global cross-sectional morphology and the internal pore architecture. Regions with larger width correspond to larger pore sizes, which contribute to reduced structural mass and enhanced energy absorption capacity. In contrast, narrower regions are associated with smaller pores, which improve local stiffness and structural stability. Moreover, the continuous and smooth variation in width suggests that the leaf vein cross-section develops a gradual geometric transition during growth, which helps alleviate stress concentration and improves the overall mechanical performance under complex loading conditions.

To further characterize the global geometric feature of the leaf vein cross-section, a curve fitting analysis was performed on the width distribution, yielding the following relationship:

(7) $$H\left(x\right)=-4.205+22.275\;\exp\left(-2\cdot {\left(\frac{x-24991}{18.771}\right)}^{2}\right)$$

The results indicate that the width variation follows a Gaussian distribution with a high coefficient of determination (R^2^ = 0.98), demonstrating that the proposed model accurately captures the geometric evolution of the cross-section. The fitted curve shows that the width reaches its maximum at approximately 25 mm and gradually decreases toward both ends, forming a typical wide-center and narrow-edge morphology. This smooth and continuous variation indicates that the leaf vein cross-section develops a stable geometric gradient during growth rather than a random structure, revealing a coupling between macroscopic geometry and microscopic pore architecture.

### 3.4. Fractal Analysis of Pore Structure in Leaf Vein Cross-Section

To quantitatively characterize the gradient fractal features of the porous structure in the Royal Water Lily leaf vein cross-section, the box-counting method was employed to calculate the local fractal dimension. In this method, the processed binary image of the cross-section was covered by square grids with side length ε, and the number of occupied grids N(ε) containing pore boundaries was counted. The fractal dimension was then determined according to:

(8) $$D=-\underset{\varepsilon \to 0}{\lim}\frac{\ln N\left(\varepsilon \right)}{\ln\varepsilon }$$

The results showed that the overall fractal dimension of the cross-section was approximately 1.510, while the local fractal dimensions of the central, transition, and peripheral regions were 1.474, 1.480, and 1.336, respectively. These findings indicate that the porous architecture does not satisfy the strict scale-invariant self-similarity required for a classical fractal system. Instead, obvious spatial variations in local fractal dimensions were observed across different regions. As the structure transitions from the central region to the peripheral region, the pore size, quantity, and spatial arrangement gradually change, exhibiting a gradient fractal-like architecture characterized by scale progression and regional heterogeneity. Such gradient porous characteristics contribute to progressive collapse and staged stress transfer during compression, thereby improving the overall energy absorption stability and buffering capability of the structure.

## 4. Development and Analysis of the Numerical Model

### 4.1. Feasibility Analysis of the Leaf Vein Cross-Section Numerical Model

In Section 3, the physical structures of leaf vein cross-sections at different sampling locations were measured and analyzed. Due to significant variations in pore size, area, and spatial distribution, the real complex geometry was parameterized and simplified to further investigate the independent effects of porous structural parameters on mechanical behavior and energy absorption performance [34]. Considering the strong randomness and coupling effects of pore size, shape, and spatial distribution in natural leaf veins, direct use of the real morphology would make it difficult to decouple the influence of individual structural parameters on the mechanical response. Therefore, based on a structural biomimetic approach, the leaf vein cross-section was idealized as a parametric model with an elliptical outer boundary and regularly distributed elliptical pores. This modeling strategy preserves the overall geometric characteristics and pore architecture while improving controllability and analytical interpretability.

During model construction, the material parameters were defined according to the constitutive behavior obtained from tensile tests of Royal Water Lily leaf vein samples reported in the literature, thereby ensuring consistency between the numerical model and the biological prototype [35]. The leaf vein material was modeled as a homogeneous isotropic elastic–plastic continuum, with a density of 5.092 × 10^−10^ t/mm^3^, Poisson’s ratio of 0.3, and Young’s modulus of 1.73 MPa. Since the present study mainly focuses on the quasi-static compressive response, progressive collapse behavior, and energy absorption characteristics of the porous structure, no explicit material damage or fracture model was introduced. Experimental observations indicated that the specimens mainly underwent large deformation and progressive local buckling during compression, without obvious brittle fracture or complete material separation. Therefore, the macroscopic mechanical response was considered to be governed primarily by geometric collapse rather than material failure. Under these conditions, the adopted elastic–plastic constitutive model was sufficient to accurately capture the load–displacement response and deformation evolution. By keeping the material properties, loading conditions, and boundary conditions constant, while only varying key geometric parameters such as porosity, pore ellipticity, and pore distribution pattern, both single-factor and multi-factor decoupling analyses of the energy absorption mechanism were achieved. This strategy preserves the essential biomimetic characteristics of the porous leaf vein structure while enabling systematic investigation of the intrinsic relationship between structural parameters and mechanical performance, thereby providing a theoretical basis for subsequent optimization of bioinspired energy-absorbing structures.

**Figure 8:**
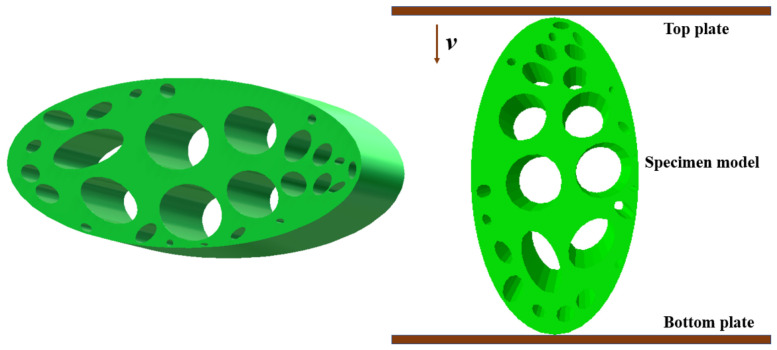
Numerical model of a cross-sectional specimen of a leaf vein.

The leaf vein cross-section was further simplified into an elliptical domain, and an approximate elliptical model was constructed using SolidWorks 2020, with overall dimensions of A × B × H = 25 mm × 14 mm × 100 mm. Based on the measured pore sizes, elliptical pores were embedded within the cross-section, arranged in a layered pattern within the A × B elliptical domain to reproduce the porous architecture of the leaf vein. A total of 27 elliptical pores were introduced to approximate the real geometric features, as shown in Figure 8.

**Figure 9:**
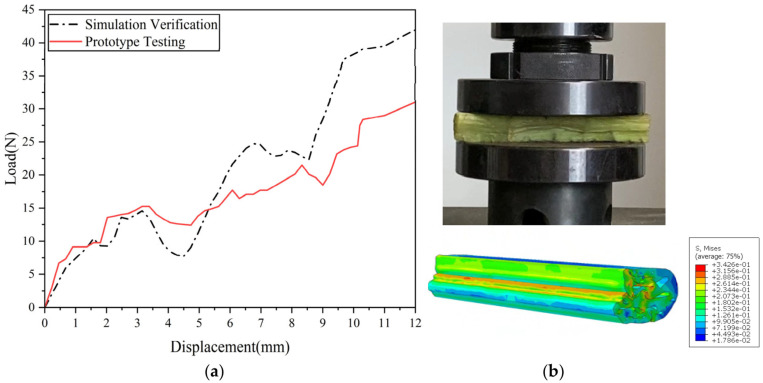
(**a**) Comparison of load–displacement curves from quasi-static compression tests on leaf vein cross-sections and simulation models; (**b**) the comparison of deformation modes.

The finite element model was discretized using reduced-integration hexahedral elements. Approximately 12–16 elements were distributed along the circumference of each elliptical pore to ensure accurate description of local stress concentration and deformation behavior. In addition, local mesh refinement was applied in the pore regions and stress concentration zones, while relatively coarser meshes were used in low-gradient regions to improve computational efficiency. The material properties were defined as follows: density 5.092 × 10^2^ kg/m^3^, Poisson’s ratio 0.3, and Young’s modulus 1.73 MPa. Rigid aluminum plates were placed above and below the specimen to ensure uniform loading, with dimensions of 120 mm × 120 mm. Their centers were aligned along the same vertical axis as the specimen. The material properties of the plates were defined as density 2.73 × 10^3^ kg/m^3^ and Young’s modulus 70 GPa [36]. Surface-to-surface contact was adopted between the plates and the porous structure, and the friction coefficient was set to 0.2 [37], which falls within the commonly reported range for biological soft materials under quasi-static compression. This value was selected to realistically represent the interfacial interaction observed in the experiments while maintaining numerical stability during large deformation contact analysis. During simulation, the bottom plate was fully constrained, while a velocity boundary condition of 2 mm/min was applied at the reference point of the upper plate. The total compression displacement was 12 mm. The resulting load–displacement curve of the leaf vein model is shown in Figure 9a.

To validate the finite element model of the porous cross-section of the Royal Water Lily leaf vein, numerical simulation results were compared with quasi-static compression experiments under identical material properties and loading conditions (Figure 9a). The load–displacement curves show good agreement in overall trends, load levels, and peak values, indicating that the model can accurately capture the macroscopic mechanical behavior. Energy absorption indices were obtained by integrating the load–displacement curves (Table 1). Although minor discrepancies exist—particularly in mean crushing force (MCF) and crush force efficiency (CFE)—these differences mainly arise from geometric irregularities, pore wall thickness variations, material heterogeneity, and simplifications in the friction coefficient, which are not fully represented in the numerical model.

Beyond the global load response, the comparison of deformation modes (Figure 9b) demonstrates that the simulation also reproduces local progressive collapse behavior observed in the experiments. Specifically, the progressive axial compression initiates from the loading region, accompanied by local pore wall buckling and gradual densification along the axial direction. The overall deformation exhibits layered collapse, and stress concentrations appear around pores, which are consistent with experimentally observed local collapse patterns. These results indicate that the finite element model can reliably predict both global mechanical response and local deformation mechanisms, confirming its suitability for subsequent parametric studies and structural optimization.

### 4.2. Effect of Porosity on the Leaf Vein Cross-Sectional Structure

To investigate the influence of porosity on the mechanical response and energy absorption characteristics of the biomimetic porous cross-section of Royal Water Lily Leaf veins, porosity was selected as a single design variable for numerical simulation. Under otherwise identical structural parameters, five porosity levels (10%, 20%, 30%, 40%, and 50%) were considered to represent different degrees of structural lightweighting and to evaluate corresponding load-bearing and energy absorption behaviors. For each case, the total pore area was determined according to the target porosity. Based on the ellipticity parameter, the major and minor axes of individual pores were calculated, enabling the construction of parameterized models with different porosity levels. This approach effectively isolates the influence of porosity on the mechanical response and provides a foundation for subsequent multi-parameter optimization. All material properties, mesh settings, boundary conditions, and loading conditions were kept consistent with those described previously. The geometric parameters of each model implemented in ABAQUS are summarized in Table 2.

**Figure 10:**
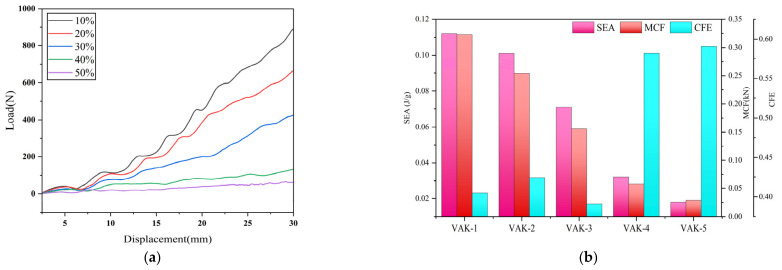
Effect of porosity on the mechanical performance of the leaf vein cross-sectional structure: (**a**) comparison of load–displacement curves; (**b**) comparison of energy absorption performance.

Based on the load–displacement curves obtained under different porosity conditions, the specific energy absorption (SEA), mean crushing force (MCF), and crushing force efficiency (CFE) were systematically evaluated. Considering that different porosity levels lead to distinct instability and collapse modes during compression, an effective compression displacement was introduced as a unified evaluation criterion to ensure a physically consistent comparison of energy absorption performance, as shown in Figure 10a,b. The results indicate that with increasing porosity, the initial stiffness of the structure decreases significantly, and the slope of the elastic stage gradually reduces. In the plateau region, structures with medium porosity (20–30%) exhibit more stable load-bearing behavior, whereas low-porosity structures show continuous strengthening without a well-defined plateau. In the densification stage, low-porosity structures exhibit a rapid increase in load-carrying capacity, while high-porosity structures show a more gradual increase. From an energy absorption perspective, MCF decreases with increasing porosity, whereas CFE shows an overall increasing trend, indicating a transition from high-strength unstable deformation to low-strength but more stable collapse behavior. SEA reaches relatively high values at low porosity, while remaining relatively stable within the 20–30% range, indicating superior overall performance in this interval. From the deformation mechanism perspective, medium-porosity structures achieve a favorable coordination among pore wall bending, local buckling, and global compression, enabling different regions to participate progressively in deformation and energy dissipation, thereby realizing stable energy absorption. During the densification stage, low-porosity structures exhibit rapid strengthening behavior because internal pores close quickly and form continuous contact skeletons, leading to a sharp increase in load. By contrast, high-porosity structures experience a more gradual collapse process due to their larger internal void space, resulting in smoother load growth.

Overall, both excessively high and low porosity values negatively affect the energy absorption efficiency and structural stability. Considering the deformation response, effective compression displacement, and energy absorption indicators, porosity is identified as a key design parameter governing the compressive and energy absorption behavior of the biomimetic leaf vein cross-section. The medium porosity range provides an optimal balance among load-bearing capacity, energy absorption efficiency, collapse stability, and engineering applicability.

### 4.3. Effect of Ellipticity on the Leaf Vein Cross-Sectional Structure

To investigate the influence of pore geometry on the in-plane compressive behavior of the biomimetic porous leaf vein structure, a single-factor parametric study was conducted by varying pore ellipticity while keeping all other parameters constant. Five models with ellipticity values of 1.0, 1.2, 1.4, 1.6, and 1.8 were established. The geometric parameters of each model are summarized in Table 3.

**Figure 11:**
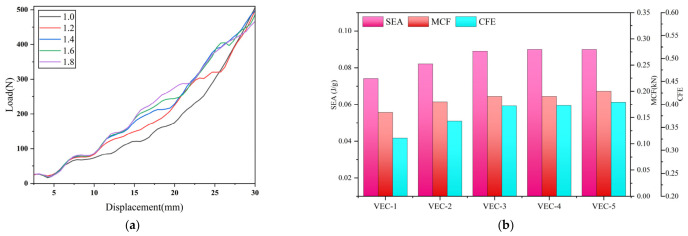
Effect of ellipticity on the mechanical performance of the leaf vein cross-sectional structure: (**a**) comparison of load–displacement curves; (**b**) comparison of energy absorption performance.

As shown in Figure 11a, the overall load–displacement response indicates that increasing ellipticity has a negligible effect on the initial stiffness; however, it significantly enhances the stability and load-bearing capacity in the plateau region, while also leading to an earlier onset of densification, thereby improving the overall energy absorption capability. The underlying mechanism of ellipticity lies in its regulation of pore wall curvature distribution, local ligament length, and load transfer pathways. At low ellipticity, the pore geometry exhibits near-isotropic characteristics, resulting in higher stiffness but limited post-buckling reconfiguration capacity. At moderate ellipticity, a favorable balance between axial compliance and lateral constraint is achieved, facilitating multi-stage cooperative collapse and sustained energy dissipation. In contrast, excessive ellipticity enhances longitudinal load transfer but weakens lateral support, leading to localized deformation and reduced deformation uniformity. Therefore, the influence of ellipticity is not monotonic but exhibits a clear optimal range behavior.

Based on the load–displacement curves under different ellipticity conditions, the specific energy absorption (SEA), mean crushing force (MCF), and crushing force efficiency (CFE) were systematically evaluated, as shown in Figure 11b. The results indicate that MCF increases monotonically with ellipticity, whereas SEA and CFE first increase and then plateau, reaching optimal performance at an ellipticity of approximately 1.4. Overall, e = 1.4 provides the best balance among all performance indicators, exhibiting relatively high load-bearing capacity, improved energy absorption capability compared with low-ellipticity structures, and superior overall energy dissipation efficiency.

### 4.4. Effect of Pore Distribution Density on the Leaf Vein Cross-Sectional Structure

To quantitatively characterize the radial distribution of pores within the leaf vein cross-section, a pore distribution density coefficient was proposed as a structural parameter. Taking the elliptical outer boundary as the reference geometry, the pores were divided into inner-layer pores and outer-layer pores. The outer contour of the model was defined as an elliptical section with dimensions of 60 mm × 40 mm. To distinguish the pore distribution regions, an imaginary inner ellipse with dimensions of 40 mm × 30 mm was introduced concentrically inside the outer contour. Pores located inside this inner ellipse were defined as inner-layer pores, whereas pores distributed between the inner ellipse and the outer boundary were defined as outer-layer pores. The pore distribution density coefficient is defined as:

(9) $$\lambda =\frac{N_{0}}{N_{i}}$$

where $N_{0}$ and $N_{i}$ represent the number of outer-layer and inner-layer pores, respectively, under the constraint of a constant total pore number. When *λ* < 1, the structure exhibits an inner-dense and outer-sparse distribution; when *λ* = 1, a uniform distribution is obtained; and when *λ* > 1, the structure exhibits an outer-dense and inner-sparse distribution. This parameter effectively captures the radial spatial organization of pores and provides a parametric basis for subsequent single-factor analysis and multi-objective response surface optimization. The geometric parameters of all simulation cases are summarized in Table 4.

**Figure 12:**
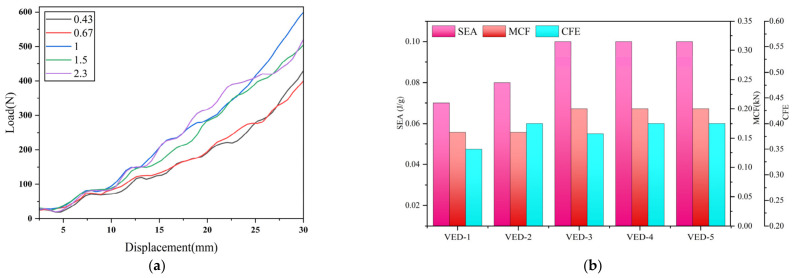
Effect of pore distribution density on the mechanical performance of the leaf vein cross-sectional structure: (**a**) comparison of load–displacement curves; (**b**) comparison of energy absorption performance.

The load–displacement response curves as shown in Figure 12a, it can be observed that all pore distribution density configurations exhibit similar elastic behavior in the initial compression stage, followed by a transition into a stable crushing plateau. However, noticeable differences arise in the loading rate and overall load-bearing capacity during the intermediate and densification stages. Overall, VED1 and VED2 exhibit relatively lower load levels in the early stage, indicating that when more pores are concentrated in the inner region, the outer skeleton remains relatively intact but lacks sufficient global cooperative load-sharing, leading to earlier onset of deformation under lower loads. As the pore distribution gradually shifts toward the outer region, VED3, VED4, and VED5 show a more pronounced increase in load-bearing capacity in the later stages, suggesting that the radial pore distribution not only influences the initial local buckling mode but also significantly alters the load transfer pathways and collapse evolution during compression. From the deformation mechanism perspective, structures with lower distribution density coefficients tend to exhibit localized instability characterized by inner-region-priority collapse, whereas higher distribution density coefficients promote cooperative load bearing and continuous energy dissipation between the inner and outer regions. This radial progressive collapse characteristic effectively alleviates local stress concentration, improves plateau-stage stability, and enhances overall energy absorption efficiency. Therefore, the distribution density coefficient fundamentally reflects the spatial organization of the internal load-transfer network and plays a critical role in regulating progressive collapse pathways and energy dissipation mechanisms.

In terms of energy absorption performance as shown in Figure 12b, the mean crushing force (MCF) increases overall with increasing pore distribution density coefficient. This is mainly attributed to the increased number of outer-layer pores, which enhances the load-bearing capacity during densification, with the most pronounced response observed at a coefficient of 2.3. The specific energy absorption (SEA) shows a trend of initial increase followed by a plateau. A significant improvement is observed when the coefficient increases from 0.43 to 1.0 or 1.5; however, a further increase to 2.3 leads to a reduced improvement or even a slight decline due to decreased mass utilization efficiency, indicating that a purely outer-biased distribution is not optimal. The crushing force efficiency (CFE) reaches optimal performance at coefficients of 1.0 and 1.5, where the plateau stage is more stable and the peak force is well matched with the mean force. In contrast, at a coefficient of 2.3, the rapid increase in load during the later stage leads to a significantly higher peak force, reducing the overall stability of the energy absorption process. Therefore, considering both energy absorption capacity and structural stability, a pore distribution density coefficient in the range of 1.0–1.5 is identified as the optimal design window.

## 5. Interaction Analysis of the Leaf Vein Cross-Sectional Numerical Model

### 5.1. Selection of Research Methodology

In practical engineering applications, multiple factors usually coexist and interact with each other. Considering only a single factor is insufficient to capture the complex coupling effects among variables [38,39]. The response surface method enables the evaluation of the influence of independent variables on response variables, including linear, quadratic, and higher-order nonlinear effects, and allows the identification of optimal parameter combinations for achieving desired performance targets [40,41]. To improve the predictive accuracy and statistical reliability of the response surface model, a three-factor, three-level response surface methodology based on the Box–Behnken Design (BBD) was adopted to optimize porosity (Vk), pore ellipticity (Vc), and pore distribution density coefficient (Vd). Compared with a full-factorial design, the BBD approach can effectively estimate quadratic response relationships and parameter interaction effects while significantly reducing the number of simulation samples. In addition, it avoids unrealistic structural configurations under extreme parameter combinations, making it particularly suitable for the multi-objective optimization of complex thin-walled porous structures. During model construction, all simulation specimens maintained identical outer boundary dimensions, total pore number, loading conditions, and boundary conditions. Only the porosity, pore ellipticity, and inner–outer pore allocation were varied to ensure direct comparability among different parameter combinations. The investigated parameter ranges were 20–40% for porosity, 1.2–1.6 for pore ellipticity, and 0.5–1.5 for the pore distribution density coefficient. These parameter levels were determined based on statistical observations of real Royal Water Lily leaf vein cross-sections and further refined according to the structural stability and manufacturability requirements identified in the preliminary single-factor analyses.

To ensure a consistent geometric basis among different parameter combinations, an equal-area principle was employed to determine pore dimensions. Specifically, under a constant total pore area, different ellipticity configurations were achieved by adjusting the ratio between the major and minor axes of the pores, thereby eliminating additional mass deviations caused by pore area variation. Meanwhile, when varying the distribution density coefficient, the total number of pores remained unchanged, and only the proportion of inner and outer pores was adjusted to generate different radial distribution patterns. This strategy ensured that the differences among models originated solely from spatial distribution characteristics rather than additional geometric scaling effects. Furthermore, considering the pronounced geometric nonlinearity and progressive collapse behavior of porous structures during compression, the response surface analysis incorporated not only specific energy absorption (SEA), but also mean crushing force (MCF) and crushing force efficiency (CFE), in order to comprehensively evaluate load-bearing capacity, energy absorption efficiency, and deformation stability. By establishing a second-order polynomial surrogate model, the linear, quadratic, and interaction effects of the design variables on the response indices could be quantitatively evaluated. Analysis of variance (ANOVA) was further conducted to assess model significance and fitting accuracy, thereby providing a reliable theoretical basis for subsequent structural optimization.

A surrogate mathematical model was developed to predict the influence of design variables on energy absorption performance. Based on the design variables and response metrics, the optimization problem in this study can be formulated as:

(10) $$\begin{array}{l} \max\left(SEA,MCF,CFE\right)\\ 20\%\leq Vk\leq 40\%\\ 1.2\leq Vc\leq 1.6\\ 0.5\leq Vd\leq 1.5 \end{array}$$

The response surface analysis was performed using Design-Expert 13 software. Porosity Vk, ellipticity Vc, and pore distribution density coefficient Vd were selected as design variables. According to the Box–Behnken central composite experimental design, a total of 17 sampling points were generated (the last five correspond to repeated trials and are therefore not repeatedly listed). Numerical models were constructed for each sampling point, and the corresponding performance indicators were calculated, as summarized in Table 5.

### 5.2. Mechanical Performance Evaluation Metrics

Based on the results in Table 5, second-order polynomial response surface models were obtained through regression fitting to describe the relationships between porosity (Vk), ellipticity (Vc), distribution density (Vd), and the response variables, including specific energy absorption (SEA), mean crushing force (MCF), and peak crushing force (PCF), as expressed below:

(11) $$\begin{array}{l} SEA=86.64-30.66V_{k}+2.81V_{c}+16.4V_{d}-2.07V_{k}\cdot V_{c}+4.11V_{k}\cdot V_{d}+\\ 1.53V_{c}\cdot V_{d}-0.7225{V_{k}}^{2}-2.88{V_{c}}^{2}+5.98{V_{d}}^{2} \end{array}$$

(12) $$\begin{array}{l} MCF=191.1-96.77V_{k}+6.58V_{c}+35.45V_{d}-5.63V_{k}\cdot V_{c}+5.38V_{k}\cdot V_{d}+\\ 3.37V_{c}\cdot V_{d}+8.46{V_{k}}^{2}-6.69{V_{c}}^{2}+13.46{V_{d}}^{2} \end{array}$$

(13) $$\begin{array}{l} CFE=0.41-0.0209V_{k}+0.0146V_{c}+0.0305V_{d}-0.0075V_{k}\cdot V_{c}+0.0163V_{k}\cdot V_{d}+\\ 0.0058V_{c}\cdot V_{d}-0.0138{V_{k}}^{2}-0.0118{V_{c}}^{2}-0.0115{V_{d}}^{2} \end{array}$$

**Figure 13:**
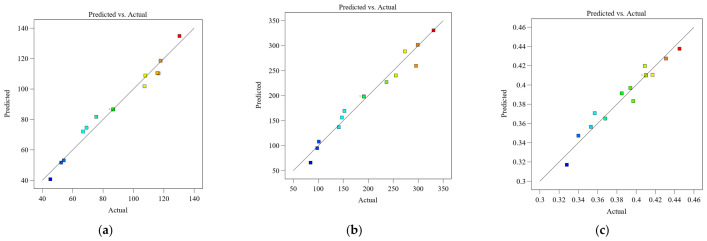
Comparison between predicted and experimental values of the three response models: (**a**) SEA; (**b**) MCF; (**c**) CFE. Comparison of predicted and actual values, with green representing lower predicted values and red representing higher predicted values. The black diagonal line indicates perfect prediction (where predicted equals actual).

Figure 13 presents a comparison between predicted and actual values for the three response variables. It can be observed that all data points are closely distributed around the line with a slope of 1, indicating strong agreement between predicted and simulated/experimental values, thereby validating the accuracy and reliability of the regression models.

Prior to analyzing response surfaces and contour plots, analysis of variance (ANOVA) was conducted to assess the significance and goodness of fit of the developed models. In this process, the F-test was employed to evaluate the overall significance of the regression models, where both the F-value and *p*-value were used to quantify statistical reliability. In general, a higher F-value and a lower *p*-value indicate a more statistically significant model. The coefficient of determination (R^2^) was used to measure the agreement between the surrogate model and the reference data; values closer to 1 indicate better predictive accuracy. As shown in Table 6, the *p*-values of all three regression models are less than 0.01. In ANOVA, the *p*-value indicates the significance of each regression coefficient on the response variable. A *p*-value below 0.05 indicates a statistically significant effect, while a *p*-value below 0.01 indicates a highly significant effect. Based on this criterion, the effects of individual factors and their interactions were statistically evaluated.

**Figure 14:**
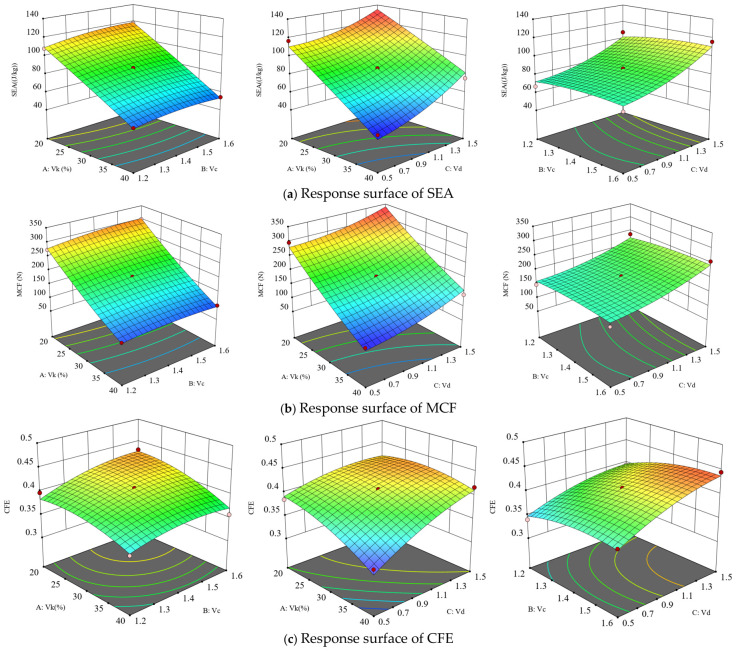
Response surfaces of the three surrogate models.

The results indicate that for SEA, Vk and Vd are the dominant influencing factors. For MCF, Vk and Vd also play leading roles. For CFE, Vk, Vc, Vd, and the interaction term Vc × Vd all exhibit significant effects. Overall, the structural performance does not vary monotonically with a single parameter but instead exhibits locally optimal behavior under coupled multi-parameter interactions. Specifically, porosity governs the overall load-bearing and energy absorption capacity, ellipticity controls local deformation patterns, and pore distribution density regulates collapse stability. The response surface results considering these parameters and their interactions are shown in Figure 14.

To validate the predictive accuracy of the response surface model, three representative numerical models with different combinations of porosity (Vk), ellipticity (Vc), and pore distribution density (Vd) were randomly selected for additional simulations. Table 7 summarizes the simulation results, predicted values, and their corresponding relative errors. The results show that all prediction errors are within 5%, demonstrating the high accuracy and reliability of the developed response surface models. Furthermore, the multi-objective optimization confirms that Vk, Vc, and Vd all significantly influence the mechanical performance of the leaf vein cross-sectional structure, and together they comprehensively characterize the structural behavior without redundancy.

**Figure 15:**
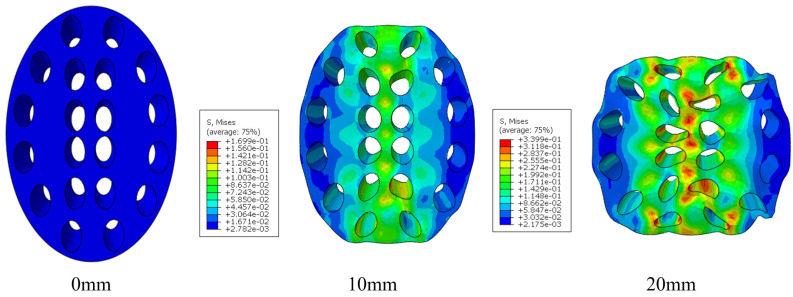
Deformation process of the 3D model of the leaf vein cross-sectional porous structure.

Based on the developed surrogate models and response surface analysis, a multiobjective optimization was conducted using the Optimization-Numerical Solutions module in Design-Expert 13 software to maximize SEA, MCF, and CFE simultaneously, with equal weighting assigned to each objective. The optimal parameter combination was identified as V_k_ =30%, V_c_ = 1.563, V_d_ = 1.5. The energy absorption parameters for this structure are as follows: $SEA_{optim}=110.65$ J/kg, $MCF_{optim}=243.68$ N, $CFE_{optim}=0.438$. This optimal solution corresponds to the ninth model in the experimental design, and the error between its predicted results and the simulation results is relatively small. From an engineering perspective, the optimized configuration (RSM-09) achieves a superior balance among structural safety, stability, and lightweight characteristics, making it a promising candidate for practical applications. As illustrated in Figure 15, the structure establishes a stable load transfer path at the early stage of compression and subsequently evolves into a continuous central collapse band. The deformation process is characterized by high symmetry and progressive collapse behavior. No significant local instability or asymmetric failure is observed throughout the compression process, indicating excellent stability and energy absorption capability, which represents an ideal deformation mode for energy-absorbing structures.

The porous structure of the Royal Water Lily Leaf vein cross-section exhibits a gradient fractal characteristic with scale progression and regional heterogeneity. In the multi-factor response surface analysis, porosity, ellipticity, and distribution density coefficient not only individually affect EA, SEA, MCF, and CFE, but their interactions also play a critical role in regulating the energy absorption performance of the structure. This indicates that the mechanical response of such structures arises from the synergistic effect of multi-scale geometric parameters rather than being governed by a single dominant factor. Under compressive loading, the porous leaf vein structure undergoes progressive collapse along the principal stress path. This inside-out deformation sequence is characterized by initial buckling of the inner pore units, which absorb the initial impact energy, followed by the sequential engagement of outer pore structures in load bearing and deformation, thereby enabling continuous buffering and stable energy dissipation. The simulated inside-out progressive collapse aligns with biological observations, wherein the leaf vein cross section exhibits a larger central pore region surrounded by progressively smaller peripheral pores. This gradient pore distribution creates a stiffness mismatch that promotes sequential load transfer from inner to outer regions, explaining the observed progressive collapse mechanism. Such deformation evolving from local to global and from inner to outer exemplifies a gradient fractal energy absorption mechanism. Consequently, the superior mechanical performance of the leaf vein porous structure stems fundamentally from the multiscale cooperative deformation enabled by its gradient fractal architecture.

## 6. Discussion

This study focuses on the porous cross-sectional structure of the Royal Water Lily Leaf vein. Based on macroscopic structural observation, mechanical testing, geometric modeling, and numerical simulation, the mechanical characteristics of its gradient fractal features and their influencing factors are systematically investigated. The results provide theoretical support and data for the optimal design of bioinspired thin-walled energy-absorbing structures.

Single-factor analysis shows that porosity, pore ellipticity, and distribution density coefficient all significantly affect the load–displacement response and energy absorption performance. As porosity increases, the overall load-bearing capacity gradually decreases, while the specific energy absorption first increases and then decreases. Changes in ellipticity mainly influence the buckling mode of pore units and the stability of the plateau stage, thereby affecting load fluctuations. The distribution density coefficient regulates the allocation of inner and outer pores, significantly influencing collapse paths and deformation coordination. These factors exhibit different mechanisms at the elastic, plateau, and densification stages, indicating a stage-dependent regulation behavior.

Multi-factor response surface analysis further demonstrates significant interactions among porosity (Vk), ellipticity (Vc), and distribution density coefficient (Vd). These interactions produce coupled effects on EA, SEA, MCF, and CFE. Compared with single-factor control, multi-parameter optimization significantly improves overall energy absorption performance. Within the studied range, the optimal combination (Vk = 30%, Vc = 1.6, Vd = 1.5) demonstrates favorable engineering potential by achieving a balanced performance in load-bearing capacity, energy absorption efficiency, and stability, with Specific Energy Absorption (SEA) reaching 115.75 J/kg, Mean Crushing Force (MCF) of 248.2 N, and Crushing Force Efficiency (CFE) of 0.445. This outcome indicates that appropriate gradient parameter matching can simultaneously enhance both energy absorption efficiency and deformation stability.

The results further indicate that the porous vein structure exhibits a gradient fractal characteristic rather than an ideal self-similar fractal geometry. Although strict mathematical self-similarity is absent, the structure presents hierarchical progression and regional heterogeneity across different spatial scales. The cooperative deformation of multi-scale pore units governs the overall mechanical response. Under compressive loading, the structure progressively collapses from the inner region toward the exterior along the principal stress transmission path, allowing pore units at different hierarchical levels to sequentially participate in load-bearing and energy dissipation. This staged deformation mechanism effectively suppresses local instability and improves energy absorption efficiency. Overall, this study establishes a quantitative relationship between gradient porous morphology and energy absorption behavior in Royal Water Lily leaf veins, and demonstrates the potential of gradient fractal-inspired structures for the development of lightweight, high-efficiency bioinspired energy-absorbing systems in transportation, protective engineering, and impact-resistant applications.

## Figures and Tables

**Table 1:** Comparison of experimental and simulation energy absorption metrics.

Specimen	SEA (mJ/g)	MCF (N)	CFE	Mass (g)
Experiment	31.16	17.34	0.560	6.68
Simulation	32.67	20.61	0.515	7.565

**Table 2:** Geometric parameters of models with varying porosity in leaf vein cross-sectional structures.

Model	Major Axis of a Single Pore (mm)	Minor Axis of a Single Pore (mm)	Area of a Single Pore (mm^2^)	Model Mass (g)
VAK-1	4.35	2.90	9.92	86.33
VAK-2	6.15	4.10	19.84	76.14
VAK-3	7.54	5.03	29.76	66.15
VAK-4	8.70	5.80	39.68	56.08
VAK-5	9.72	6.48	49.60	46.09

**Table 3:** Geometric parameters of specimens with varying axis of elliptical pores.

Model	Major Axis of a Single Pore (mm)	Minor Axis of a Single Pore (mm)	Ellipticity	Model Mass (g)
VEC-1	6	6	1.0	67.75
VEC-2	6.573	5.477	1.2	67.75
VEC-3	7.099	5.071	1.4	67.75
VEC-4	7.589	4.743	1.6	67.75
VEC-5	8.050	4.472	1.8	67.75

**Table 4:** Geometric parameters of specimens with varying pore distribution configurations.

Model	Distribution Pattern (Outer/Inner)	Distribution Density Coefficient	Classification Description
VED-1	6/14	0.43	Inner-dense, outer-sparse
VED-2	8/12	0.67	Slightly inner-dense
VED-3	10/10	1	Uniform distribution
VED-4	12/8	1.5	Slightly outer-dense
VED-5	14/6	2.3	Outer-dense, inner-sparse

Note: VED1–VED5 denote five pore distribution schemes evolving from inner-dense/outer-sparse to outer-dense/inner-sparse configurations. In all cases, the total number of pores, porosity, ellipticity, and material properties remain constant, while only the allocation of pores between inner and outer regions is varied.

**Table 5:** Simulation parameters and results of the central composite design experiments.

Number	Porosity/ (%)	Ellipticity	Distribution Density Coefficient	EA (J)	SEA (J/kg)	MCF (N)	CFE
RSM-01	20	1.6	1	8.973	117.85	299.1	0.431
RSM-02	20	1.2	1	8.201	107.83	273.4	0.397
RSM-03	20	1.4	1.5	9.922	130.31	330.7	0.409
RSM-04	20	1.4	0.5	8.872	116.52	295.7	0.385
RSM-05	30	1.2	1.5	7.099	107.32	236.6	0.394
RSM-06	30	1.4	1	5.731	86.64	191.1	0.410
RSM-07	30	1.6	0.5	4.571	69.10	152.4	0.368
RSM-08	30	1.2	0.5	4.419	66.8	147.3	0.340
RSM-09	30	1.6	1.5	7.657	115.75	248.2	0.445
RSM-10	40	1.4	1.5	4.234	75.50	141.1	0.417
RSM-11	40	1.6	1	3.034	54.10	101.1	0.357
RSM-12	40	1.2	1	2.937	52.37	97.9	0.353
RSM-13	40	1.4	0.5	2.539	45.27	84.6	0.328

**Table 6:** Variance of the surrogate model.

Energy Absorption Indices	F	*p*	R^2^
SEA	32.61	<0.0001	0.9767
MCF	49.64	<0.0001	0.9846
CFE	14.91	<0.0001	0.9504

**Table 7:** Model validation.

Number	Variable	Value		SEA (J/kg)	MCF (N)	CFE
1	V_k_	27.5%	Simulation	92.42	215.23	0.412
	V_c_	1.24	Prediction	95.37	217.6	0.400
	V_d_	1.17	Error (%)	3.19	1.1	2.91
2	V_k_	30.7%	Simulation	76.58	165.41	0.381
	V_c_	1.25	Prediction	73.61	160.6	0.370
	V_d_	0.7	Error (%)	3.87	2.9	2.89
3	V_k_	37.5%	Simulation	53.51	104.2	0.358
	V_c_	1.37	Prediction	51.14	99.70	0.343
	V_d_	0.59	Error (%)	4.42	4.32	4.19

## Data Availability

The data presented in this study are available upon request from the corresponding author.
